# Supplementary material for: Causal Relationship between Aging and Anorexia Nervosa: A White-Matter-Microstructure-Mediated Mendelian Randomization Analysis
Source: Biomedicines. 2024 Aug 16;12(8):1874. doi: 10.3390/biomedicines12081874 (PMC11351342; doi:10.3390/biomedicines12081874)
Supplement: Supplementary file 1 [file biomedicines-12-01874-s001.zip › Supplementary Figure/Figure S8/Leaveoneout plot of MR analysis for TL to ubm-b-1488.pdf]

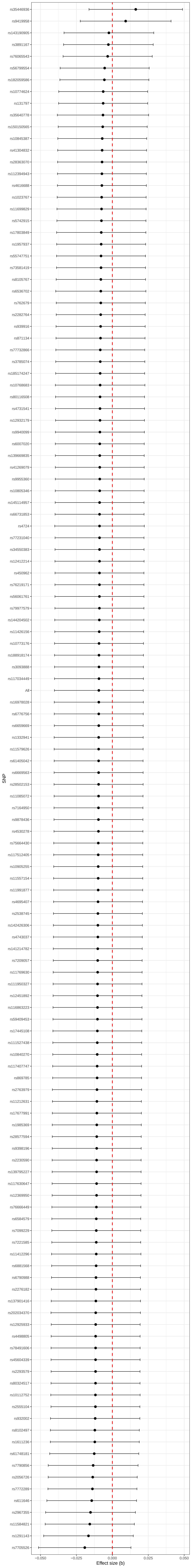

SNP
Effect size (b)
-0.050
-0.025
0.000
0.025
0.050
rs35446936
rs9419958
rs143190905
rs3891167
rs76065543
rs56799554
rs182059586
rs10774624
rs131797
rs35640778
rs150150565
rs10845387
rs41304832
rs28363070
rs112394943
rs4616688
rs1023767
rs11699829
rs5742915
rs17803849
rs1957937
rs55747751
rs73581419
rs8105767
rs6536702
rs762679
rs2282764
rs939916
rs871134
rs77732866
rs3785074
rs185174247
rs10768683
rs80116508
rs4731541
rs12932179
rs9940099
rs6007020
rs139669835
rs41269079
rs9955360
rs10805346
rs145114957
rs66731853
rs4724
rs77231040
rs34550383
rs12412214
rs450962
rs76219171
rs56061761
rs79977579
rs144204502
rs11426156
rs10773176
rs188918174
rs3093888
rs117034449
All
rs16978028
rs6776756
rs6659669
rs1332941
rs11579626
rs61405042
rs6669563
rs28502153
rs11085072
rs7164950
rs9878436
rs4530278
rs75664430
rs117512405
rs10905255
rs11557154
rs11991877
rs4695407
rs2538745
rs142426306
rs4743037
rs141214782
rs7209057
rs11769630
rs111950327
rs12451892
rs116863223
rs59409453
rs17445108
rs111527438
rs10840270
rs117407747
rs869785
rs2763979
rs11212631
rs17677991
rs1985369
rs28577594
rs9398196
rs2230590
rs139795227
rs117630647
rs12369950
rs76666449
rs6584579
rs7099229
rs7221585
rs11412296
rs6881568
rs6790988
rs2276182
rs137901416
rs202034370
rs12925933
rs4498805
rs78491606
rs45604339
rs2293579
rs80324517
rs10112752
rs2555104
rs932002
rs8102497
rs1611236
rs61748181
rs7790856
rs2056726
rs7772289
rs611646
rs2967355
rs11584821
rs1291143
rs7705526
